# Chronic Hyperplastic Candidiasis on the Tongue in a 20-Year-Old Male: A Case Report

**DOI:** 10.7759/cureus.116295

**Published:** 2026-09-15

**Authors:** Karthik Rajaram Mohan, Ramachandra Reddy Gowda Venkatesha

**Affiliations:** 1 Oral Medicine and Radiology, Vinayaka Mission's Sankarachariyar Dental College, Vinayaka Mission's Research Foundation (Deemed to be University), Salem, IND

**Keywords:** candidosis, oral candidiasis, oral thrush, pdt - photo dynamic therapy, xerostomia

## Abstract

The oral mucosa is commonly affected by oral candidiasis, an extremely prevalent fungal infection. Chronic hyperplastic candidiasis is a type of chronic mucosal lesion, characterized by white, firm, adherent plaques on the oral mucosa. In this report, we present a case of chronic hyperplastic candidiasis on the tongue, exploring its predisposing factors, clinical features, potential for malignant transformation, and treatment options.

## Introduction

Hyperplastic candidiasis is characterized by persistent white plaques on the lips, tongue, and buccal mucosa. These plaques are firm and non-scrapable [1]. Depending on their type, they can be homogeneous or nodular and may persist for many years [2]. The pathogenesis of candidal lesions is primarily mediated by the yeast Candida albicans [2]. While Candida is a normal component of the oral microflora, Candida albicans is carried by approximately 30% to 50% of people [3]. When the oral environment becomes favorable, the normally commensal Candida can flourish and become pathogenic [4]. The main clinical sign of oral candidiasis is the appearance of curdy white patches on the oral mucous membrane [5]. Immunocompromised individuals are the most likely to develop candidiasis as a secondary infection [6]. Candidiasis is also known as moniliasis, thrush, and candidosis [7]. Typically, these infections are found in the oral cavity, digestive tract, vaginal canal, and other parts of the body [8]. For Candida to become pathogenic, a favorable environment is necessary [9]. In severe cases, surgery may be required [9]. Treatment usually involves antifungals [10].

## Case presentation

A 20-year-old male reported to our department for alteration in taste. There was no history of decreased food intake. His past medical history included typhoid one month prior, for which he received oral ciprofloxacin, a fluoroquinolone antibiotic, at a dosage of 500 mg twice daily for one week. Following this treatment, he experienced an alteration in taste and visited our department. The patient reported no known drug allergies and no deleterious habits such as chewing or smoking tobacco. On intraoral examination, a white homogeneous plaque was observed on the posterior one-third of the tongue's dorsum, measuring approximately 1.5 x 2.5 cm, located in front of the the circumvallate papilla region. No similar lesions were found in other anatomical regions of his oral cavity. His oral hygiene was satisfactory. The lesion was non-scrapable, non-tender, and leathery in texture. Based on these findings, chronic hyperplastic candidiasis was provisionally diagnosed (Figure 1).

**Figure 1:**
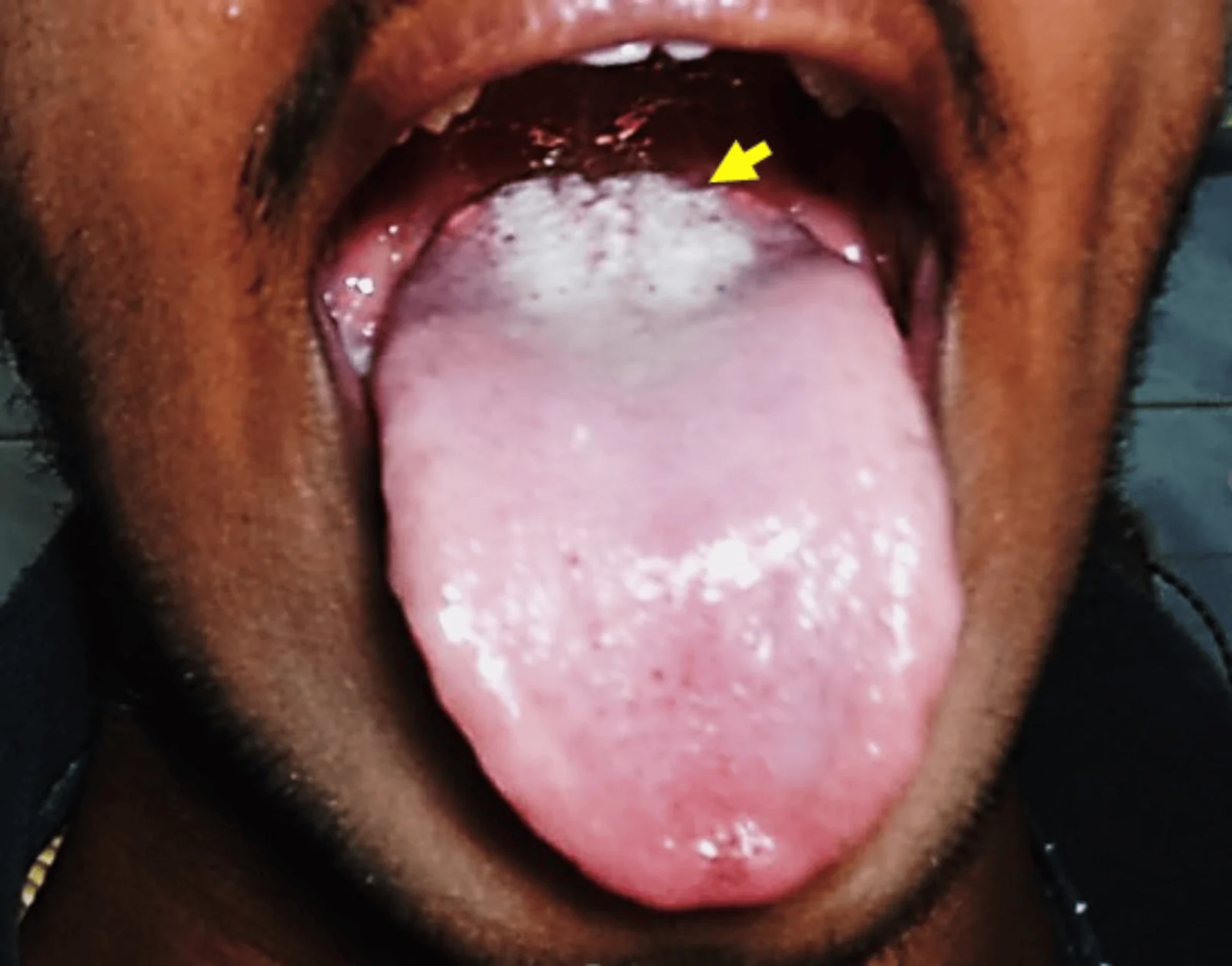
Intraoral examination revealed a white, firm, adherent, homogeneous plaque on the posterior one-third of the dorsum of the tongue, in front of the circumvallate papillae.

The differential diagnosis included lichen planus and immunosuppressive diseases such as uncontrolled diabetes and HIV-induced oral candidiasis. However, our patient had a glycated hemoglobin (HbA1C) level of 4.7% and a random blood glucose level of 136 mg/dl, and he was seronegative for HIV. The characteristic cotton fluffy appearance of lichen planus on the tongue's dorsum was not present in our patient.

For treatment, Sporanox (Janssen Pharmaceuticals) capsules containing Itraconazole 200 mg were prescribed once daily for four weeks, as it is more potent than fluconazole and its absorption is not affected by food intake. Additionally, Tablet Zincovit (Apex Laboratories Pvt Ltd), providing 10 mg of zinc, was prescribed once daily for four weeks to treat dysgeusia.

Clinical significance

Chronic hyperplastic candidiasis on the dorsum of the tongue in a 20-year-old adult male, which occurred following intake of oral ciprofloxacin antibiotic medication for typhoid, although his oral hygiene was satisfactory, and there were no associated comorbidities like diabetes or HIV. The literature revealed that it occurs only in the advanced age group above 50.

Limitations

Investigations such as a smear, Periodic Acid-Schiff (PAS) stain, and culture were not performed to identify the candida species causing the non-scrapable white lesion on the tongue. The patient, hailing from a remote place, did not report for follow-up.

## Discussion

CHC is a chronic mucosal lesion characterized by nodular or speckled hyperplasia [6]. It is more commonly seen in males and typically occurs in individuals aged 55.5 years and older. However, in our case, we reported its occurrence in a 20-year-old young male. Iwamuro M et al. described discrete multiple candidal white plaques in the esophagus, oral cavity, larynx, and pharynx during esophagogastroduodenoscopy in a 63-year-old female with hypertension and chronic thyroiditis [7]. Patients who are chronic smokers, have diabetes or have undergone transplant surgery are at risk of developing CHC, a specific clinical subtype of oral candidiasis characterized by whitish oral plaques that cannot be wiped away [8]. Approximately 1.61% of patients are infected due to poor oral and denture hygiene, xerostomia, iron deficiency anemia, persistent antibiotic use, steroid therapy, radiation or chemotherapy for oral cancer, and immunosuppression caused by conditions such as diabetes, HIV, and systemic diseases like leukemia [9,10].

Malignant transformation potential

A study by Zhang W et al. found that among oral CHC patients, the male-to-female ratio was approximately three to two, and the average age at diagnosis was around 55. They reported that 21% of patients with CHC exhibited dysplasia, with two cases eventually developing into cancer [11]. According to Lorenzo-Pouso AI et al., 12.1% of CHC patients experienced malignant transformation [12]. Given its polymorphic clinical presentation and potential for malignant transformation, CHC presents challenges in diagnosis and management [13]. Li B et al. reported a case of CHC treated with photodynamic therapy using 5-aminolevulinic acid [14].

## Conclusions

CHC is a neglected clinical condition but is considered clinically important due to its potential for malignancy. One must be aware of these clinical features and predisposing factors, as they aid in early diagnosis and help prevent epithelial dysplasia, thereby improving the quality of life in affected patients.
